# Supplementary material for: Modeling glioblastoma heterogeneity as a dynamic network of cell states
Source: Mol Syst Biol. 2021 Sep 16;17(9):e10105. doi: 10.15252/msb.202010105 (PMC8444284; doi:10.15252/msb.202010105)
Supplement: Supplementary file 6 — Source Data for Figure 5 [file MSB-17-e10105-s004.zip › Figure5A_sourcedata/GSEA_3017/hallmarks_stateD.GseaPreranked.1621934542601/HALLMARK_ESTROGEN_RESPONSE_LATE.html]

Details for gene set HALLMARK\_ESTROGEN\_RESPONSE\_LATE[GSEA]

|  || Dataset | state23017 |
| Phenotype | NoPhenotypeAvailable |
| Upregulated in class | na\_neg |
| GeneSet | HALLMARK\_ESTROGEN\_RESPONSE\_LATE |
| Enrichment Score (ES) | -0.3693357 |
| Normalized Enrichment Score (NES) | -1.5324724 |
| Nominal p-value | 0.062569834 |
| FDR q-value | 0.16513966 |
| FWER p-Value | 0.804 |
Table: GSEA Results Summary

  

Fig 1: Enrichment plot: HALLMARK\_ESTROGEN\_RESPONSE\_LATE      
 Profile of the Running ES Score & Positions of GeneSet Members on the Rank Ordered List

  

| PROBE | GENE SYMBOL | GENE\_TITLE | RANK IN GENE LIST | RANK METRIC SCORE | RUNNING ES | CORE ENRICHMENT || 1 | FABP5 |  |  | 1 | 0.885 | 0.0671 | No |
| 2 | CELSR2 |  |  | 220 | -0.255 | -0.0819 | No |
| 3 | HOMER2 |  |  | 446 | -0.279 | -0.2345 | No |
| 4 | CPE |  |  | 482 | -0.283 | -0.2399 | No |
| 5 | PRSS23 |  |  | 498 | -0.284 | -0.2297 | No |
| 6 | CKB |  |  | 554 | -0.292 | -0.2498 | No |
| 7 | BTG3 |  |  | 581 | -0.296 | -0.2472 | No |
| 8 | NCOR2 |  |  | 628 | -0.306 | -0.2593 | No |
| 9 | ADD3 |  |  | 663 | -0.312 | -0.2616 | No |
| 10 | RAB31 |  |  | 667 | -0.313 | -0.2400 | No |
| 11 | PLK4 |  |  | 699 | -0.319 | -0.2394 | No |
| 12 | CDC20 |  |  | 793 | -0.341 | -0.2852 | No |
| 13 | MEST |  |  | 864 | -0.360 | -0.3118 | No |
| 14 | STIL |  |  | 888 | -0.369 | -0.3013 | No |
| 15 | MOCS2 |  |  | 977 | -0.407 | -0.3382 | Yes |
| 16 | PRKAR2B |  |  | 1005 | -0.416 | -0.3271 | Yes |
| 17 | CDC6 |  |  | 1008 | -0.418 | -0.2966 | Yes |
| 18 | TSPAN13 |  |  | 1046 | -0.443 | -0.2912 | Yes |
| 19 | ASCL1 |  |  | 1097 | -0.467 | -0.2940 | Yes |
| 20 | ALDH3A2 |  |  | 1123 | -0.489 | -0.2759 | Yes |
| 21 | EMP2 |  |  | 1124 | -0.489 | -0.2384 | Yes |
| 22 | RBBP8 |  |  | 1128 | -0.494 | -0.2028 | Yes |
| 23 | HSPA4L |  |  | 1131 | -0.497 | -0.1663 | Yes |
| 24 | OLFM1 |  |  | 1136 | -0.500 | -0.1310 | Yes |
| 25 | IDH2 |  |  | 1146 | -0.508 | -0.0990 | Yes |
| 26 | CHPT1 |  |  | 1257 | -0.725 | -0.1284 | Yes |
| 27 | CD44 |  |  | 1270 | -0.762 | -0.0793 | Yes |
| 28 | TOP2A |  |  | 1313 | -1.528 | 0.0054 | Yes |
Table: GSEA details [plain text format]

  

Fig 2: HALLMARK\_ESTROGEN\_RESPONSE\_LATE: Random ES distribution      
 Gene set null distribution of ES for **HALLMARK\_ESTROGEN\_RESPONSE\_LATE**
